# Effects of Body Condition Score Changes During Peripartum on the Postpartum Health and Production Performance of Primiparous Dairy Cows

**DOI:** 10.3390/ani9121159

**Published:** 2019-12-17

**Authors:** Yujie Wang, Pengju Huo, Yukun Sun, Yonggen Zhang

**Affiliations:** College of Animal Science and Technology, Northeast Agricultural University, Harbin 150030, China; wangyjanet@163.com (Y.W.); huopengju@163.com (P.H.);

**Keywords:** body condition score, peripartum, fat mobilization, primiparous dairy cow

## Abstract

**Simple Summary:**

This study systematically describes the effects of body condition score (BCS) changes in primiparous cows during the peripartum period on hormone indexes, health, and production. The BCS and its changes indirectly measure the degree of fat mobilization and is a good predictor of the risk of postpartum disease. In production practice, confounding the management of primiparous and multiparous cow risks neglecting the postpartum characteristics of primiparous cows. A prospective observational study observed that primiparous cows that have a lower BCS have higher non-esterified fatty acids (NEFA) and β-hydroxybutyrate (BHBA) concentrations and more dramatic hormonal changes. Prepartum BCS changes were inconsistent and small, while after calving, there was a drastic decline in the BCS, suggesting that even a slight drop in the prepartum BCS may be a warning of a postpartum risk for primiparous cows. It is suggested that operators attach importance to the primiparous cow prepartum BCS and keep it stable through prepartum management adjustments, since an ideal BCS at calving reduces the incidence of postpartum disease.

**Abstract:**

This is a prospective observational study that evaluates the effects of body condition score (BCS) changes in primiparous Holstein cows during peripartum on their NEFA and BHBA concentrations, hormone levels, postpartum health, and production performance. The cows under study (*n* = 213) were assessed to determine their BCS (5-point scale; 0.25-point increment) once a week during the whole peripartum by the same researchers; backfat was used for corrections. Blood samples were collected 21 and 7 days before calving and 7, 21, and 35 days after calving, and were assayed for NEFA, BHBA, growth hormone (GH), insulin, leptin, and adiponectin concentrations. The incidence of disease and milk yield were recorded until 84 days after calving. Cows were classified according to their BCS changes during peripartum as follows: Those that gained BCS (G; ΔBCS ≥ 0.25), maintained BCS (M; ΔBCS = 0–0.25), or lost BCS (L; ΔBCS ≥ 0.5). The BCS at −21 days and at 7, 14, and 21 days were different *(p* < 0.01), but trended toward uniformity in all groups at calving. The L group had higher NEFA and BHBA concentrations and hormone levels (*p* < 0.01) than the M and G groups at 21 and 35 days after calving, and had a higher incidence of uterine and metabolic diseases; however, there were no differences in production performance between the various groups. In conclusion, a lower BCS in primiparous cows during peripartum influences the NEFA and BHBA concentrations, hormone levels, and occurrence of health problems postpartum. The postpartum effects of BCS changes appear prior to calving.

## 1. Introduction

For a cow approaching calving, the periparturient period, from three weeks prepartum to three weeks postpartum, is an important stage that determines whether milk yield and dry matter intake (DMI) will rapidly increase postpartum; recovery of postpartum DMI is an important measure for avoiding a negative energy balance (NEB) [1,2]. Numerous metabolic and hormonal changes, together with a series of stress reactions, such as calving, lactating, and ration changes, involving feeding management during this period, have a direct effect on the health, reproduction, and lactation performance of cows [3,4]. Parity is a well-known risk factor for disease: Multiparous cows are more likely to develop ketosis and hypocalcemia [5,6]; the evolution of metabolic profiles in healthy and sick cows during the periparturient period varies according to parity [7]. Studies have found that primiparous cows have higher concentrations of insulin-like growth factor-I, lower concentrations of BHBA throughout periparturient, and higher concentrations of leptin; these differences are associated with significantly lower milk production and body condition scores [8,9]. These results suggest that the management of primiparous cows during the periparturient period should be different.

Body condition score (BCS) is strongly correlated with energy reserves, directly reflecting the fat reserves of individual dairy cows. Changes in the BCS rather than a single BCS measurement, which is frequently used to monitor energy balance as a practical tool for dairy farm management, are widely used and easy to determine [10]. The peripartum BCS and a series of changes, including the BCS at calving and the rate and degree of BCS reduction after calving, may indicate the increase of non-esterified fatty acids, possibility of postpartum diseases and differences in production performance. Studies have shown that the BCS of multiparous cows can be regarded as a prediction tool due to the strong association between the BCS and metabolic diseases, including hepatic lipidosis, ketosis, and abomasum displacement [11,12,13]. The main reason for this strong association is that weight loss over 50 kg due to improper prepartum feeding significantly inhibits DMI and milk production; meanwhile, high milk production and the consequent synthesis of milk fat result in a high degree of fat mobilization, causing cows to go through NEB [14]. Health and performance at the primiparous stage have a profound impact on later stage incidence of disease and production potential; thus, reasonable management of cows’ body condition is particularly important. Little is known about the characteristics and the reference range of the BCS in primiparous cows on disease prediction, hormonal levels, and lactation performance. Considering that primiparous cows represent a high proportion of cows in production, it is particularly necessary to further study the prepartum BCS of primiparous dairy cows and to use a hormonal index to specifically investigate the influence of BCS changes on fat metabolic, health, and lactation.

Adipose tissue reserves are predominantly controlled by the energy balance and abundance of insulin, with the expression and tissue responsiveness of key hormones being altered to maintain physiological equilibrium at the beginning of chronic energy deficiency [15]. Growth hormone (GH) directly regulates ruminant adipose stores. Insulin is an antagonist of the lipolytic actions of GH and lowers mobilization of the tissue reserves. Adiponectin is recognized to play an important role in metabolic syndrome. Leptin serves as an intake satiety signal by predominantly acting on the brain [10]. The somatotropic axis, primarily consisting of growth hormone (GH; somatotropin) and insulin-like growth factor-I (IGF-I; somatomedin), is essential for the regulation of intrahepatic lipid metabolism [16]. Association between peripartum BCS, fat mobilization, and postpartum serum insulin concentration has been demonstrated in several studies [17,18,19,20]; besides, an increase in serum concentrations of NEFA and BHBA, as well as decreased serum concentrations of insulin and glucose, are indicators of NEB [21]. Studies convincingly demonstrate that exogenous bovine somatotropin (BST) results in an increase in milk yield in treated animals and results in a series of coordinated adaptations in their body tissues to support the increased use of nutrients for milk synthesis [22]. A recent study exploring the association between postpartum plasma insulin and NEFA and BHBA concentrations demonstrated that cows with low plasma insulin had significantly higher concentrations of circulating NEFA; moreover, cows with low plasma insulin during early postpartum produced more milk and had higher FCM (fat corrected milk) or ECM (energy corrected milk) compared with cows with high plasma insulin [23]. The serum adiponectin concentration was positively associated with the insulin responsiveness of glucose and NEFA metabolism [24]. Most of the studies that investigated hormone concentration in the context of fat and glucose metabolism used multiparous cows in their experiments; however, differences and changes in fat metabolic hormone levels among primiparous cows, especially the direct effects mechanism, are not fully understood and deserve further investigation.

This study highlights the need for the preferential treatment of primiparous cows to ensure that their BCS trajectory is sufficient for calving, and the need to adopt a management strategy for adjusting the prepartum BCS to maximize the prevention of postpartum disease and production potential during later stages. Furthermore, it was hypothesized that testing the levels of the key regulatory hormones related to fat mobilization would result in a better understanding of the factors that influence BCS mobilization and replenishment. The objectives of the current study were to evaluate the association between BCS changes and hormone levels during peripartum and postpartum with the health and performance characteristics of primiparous lactating Holstein cows, and to examine the herd- and cow-level factors that influence the BCS profile, thus promoting animal management aimed at improving farm productivity, profit, and animal welfare.

## 2. Materials and Methods

### 2.1. Animals and Management

This prospective observational experiment was conducted on a commercial farm in Harbin City, Heilongjiang Province, China from August 2018 to January 2019. Two hundred and thirteen primiparous cows from a total of 692 lactation cows met the enrollment criteria and were used in the current study. All cows were synchronized using a Double-Ovsynch protocol for first TAI (Timed Artificial Insemination) with a progesterone implant during the Ovsynch. Protocol of synchronization started when cows had 60–65 days in milk. Primiparous cows entered the peripartum period during August and September. The calving date was synchronized to be in fall, during September and October; thus, there was no calving month effect. Milk yield data were collected from 5 to 84 days after calving. Disease records were kept to 84 days postpartum. The collection of postpartum data was completed in January. The living environment of the experimental primiparous Holstein cows (*n* = 213) during peripartum was consistent: Cows were housed in free-stall barns that included mattresses composed of rice hulls, and were equipped with self-locking head gates at the feed line and with a cross-ventilation system with fans and spray devices. One to 14 days after calving, cows were relocated to the fresh-cows cowshed for postpartum care. Cows were milked three times a day at 7:00 a.m., 2:30 p.m., and 10:30 p.m. The milk yield of individual cows was recorded and stored in the software of the automatic milking system. Cows were fed total mixed ration (TMR) formulated according to NRC (2001) to meet the nutritional requirements of each period (pre- and postpartum). Prepartum cows were fed beginning at 4 p.m., fresh cows were fed at 7:35 a.m., with ad libitum access to food and water. All cows were synchronized using a Double-Ovsynch protocol for the first TAI with a progesterone implant during Ovsynch. The synchronization protocol was started when cows were in milk for 60–65 days.

### 2.2. Collection, Treatment, and Assessment of Blood Samples

Blood samples were collected throughout the experimental period relative to parturition at −21, −7, 7, 21, and 35 days from coccygeal vessels using a sterile syringe prior to the morning feeding. The samples were collected in a 5 mL evacuated centrifuge tube containing heparin anticoagulant and centrifuged at 2000× *g* for 15 min. Supernatant was collected, aliquoted into 1.5 mL centrifuge tubes, and stored at −20 °C for the measurement of blood biochemical and hormonal indexes, including NEFA, BHBA, GH, insulin, adiponectin, and leptin. The analyses were performed using commercial kits (Jiang Lai biotechnology co. LTD, Shanghai, China) by the enzymatic colorimetric endpoint method. The values were recorded using a Switzerland Tecan multifunctional microplate reader.

### 2.3. Assessment of the BCS and Classification

The BCS were recorded at the beginning of peripartum (21 d before calving) and scored weekly throughout peripartum at 21, 14, and 7 days before calving; the day of calving; and 7, 14, and 21 days after calving. The BCS assessment was completed by a doctor and a master trained by the College of Animal Science and Technology, Northeast Agricultural University. These personnel had also completed four-level course training, had practical operation experience in a commercial farm, and used the visual and tactile technique to determine the BCS based on the US 5-point system with a 0.25 increment, with 1 being too thin and 5 being too obese. Each cow was evaluated on each occasion by both researchers. Before each morning feeding, cows were kept in a normal standing posture [25]. To guarantee the accuracy and objectivity of the final BCS, a portable ultrasound backfat instrument was used to measure the fat thickness of the rump at each BCS assessment, and developed a linear regression model of BCS according to US 5-point system rules to determine BCS. The maximum penetration depth of the backfat instrument probe was 10 cm. The probe was placed vertically at 1/4 to 1/5 of the line connecting the ischial tuberosity and the hip tuberosity, and all values were measured on the right side. The final BCS of each cow was calculated using the average of three data points. Cows were classified according to BCS changes during peripartum (BCS at 21 days minus BCS at −21 days). The classifications included: Gained BCS (Gained; ΔBCS > 0), maintained BCS (Maintained; −0.25 ≤ ΔBCS ≤ 0), and lost BCS (Lost; ΔBCS < −0.25).

### 2.4. Disease Definition

Data on health status (mastitis, metabolic and digestive disorders, and metritis) were collected from the day of calving to 84 days in milk. Early warning of disease was based on a system that was fitted to the cows: A neck-mounted electronic rumination and activity monitoring tag (HR Tags, SCR Dairy, Netanya, Israel). At all times, cows that the tag identified were examined to establish a preliminary diagnosis by the same personnel. Information on the treatment and collected data were stored on the on-farm software. Fresh cows were observed daily from calving to 14 days postpartum until relocation to a cowshed for healthy cows. Cows with health disorders were subjected to required milk withdrawal and placed in a separate pen, and their milk was discarded until it became saleable. The clinical examination included a direct observation (general appearance and attitude, muscle strength, presence of fetal membranes outside the vulva, evaluation of vaginal discharge, foot health, udder health, and manure consistency), rectal temperature, urinary ketones, and rumen auscultation.

Cows were evaluated for metritis postpartum by palpation. Metritis was characterized by an enlarged uterus with a fetid watery red–brown discharge within 21 days postpartum. The rectal temperature was measured for cows with metritis, and those with a temperature of 39.5 °C were diagnosed with puerperal metritis. Abortion was defined as failure to deliver a normal calf. Retained fetal membrane was defined as failure to detach fetal membranes within 24 h postpartum. At every milking, all cows were examined for signs of clinical mastitis by the herd personnel immediately before milking: Clinical mastitis was characterized by the presence of abnormal milk or by signs of inflammation in one or more quarters. The herd personnel milked three handfuls and checked whether characterized by the presence of abnormal milk. A case of milk fever was defined as a prostrated cow with minimal rumen contractions that responded to an intravenous calcium treatment within 30 min. Cows with a decreased appetite and altered patterns of milk production had their urine tested for ketone bodies (Keto-Stix, Bayer Diagnostics, Tarrytown, NY, USA), and those that tested at or above moderate were diagnosed with ketosis. Cows with a metallic (ping) sound at percussion auscultation of the left or right abdomen (between the 4th and 13th ribs) were diagnosed with a displacement of the abomasum. Cows with scant manure, lack of appetite, and rumen stasis were diagnosed with indigestion. Respiratory disease was characterized by panting, rectal temperatures >39.5 °C, crackling, rales, or percussion dullness when auscultating the lungs. Cows with traumatic events (cesarean section, udder/teat cuts, and broken limbs) were excluded from the study. Occurrences of retained fetal membranes, abortion, and metritis were grouped into one variable: Uterine disease. Milk fever, ketosis, and displacement of the abomasum were grouped into one variable: Metabolic disease. Cows with a diagnosis of respiratory disease and cows with undefined sickness were grouped into the category: Other diseases.

### 2.5. Statistical Analysis

This study was a prospective observational study. The BCS at various times and the NEFA, BHBA, GH, insulin, leptin, and adiponectin concentrations were analyzed by GLM using MIXED PROC using SPSS software (version 22.0, IBM SPSS Statistics, Chicago, state of Illinois, USA). The model included the fixed effects of the experimental group (G, M, or L), the fixed effect of week in lactation, and interaction of the groups by week in lactation. A BCS of −21 days was used as covariance. To verify significant differences between the groups, data were analyzed using ANOVA in SPSS. Milk yield data were processed using ANOVA in SPSS. Significance was declared at *p* < 0.05 unless otherwise indicated. The distribution of the BCS in cows between various groups at day −21 was processed using Microsoft Office Excel. Health event statistics were recorded using Microsoft Office Excel (version MSO 16.0, Microsoft, Redmond, state of Washington, USA).

## 3. Results

Primiparous cows were divided into three groups based on changes in the prepartum BCS: Gained BCS (Gained, G), maintained BCS (Maintained, M), and lost BCS (Lost, L); the proportions of each group were 15.96% (34/213), 30.99% (66/213), and 53.05% (113/213), respectively. The BCS at −21 days were different; the mean BCS (± SEM) at −21 d were 3.09 ± 0.06, 3.39 ± 0.03, and 3.45 ± 0.02 for the G, M, and L groups, respectively. The L group had the highest BCS (3.45), followed by the M group (3.39). The G group (3.09) had a mean BCS lower than the other groups (*p* < 0.01; Table 1). Cows had similar BCS on days −14, −7, and 0. However, the postpartum BCS were different from the prepartum BCS: The L group had the lowest BCS versus the BCS of the other two groups (*p* < 0.01) at 7 days. The BCS at 14 and 21 days had very significant differences between groups.

**Figure 1:**
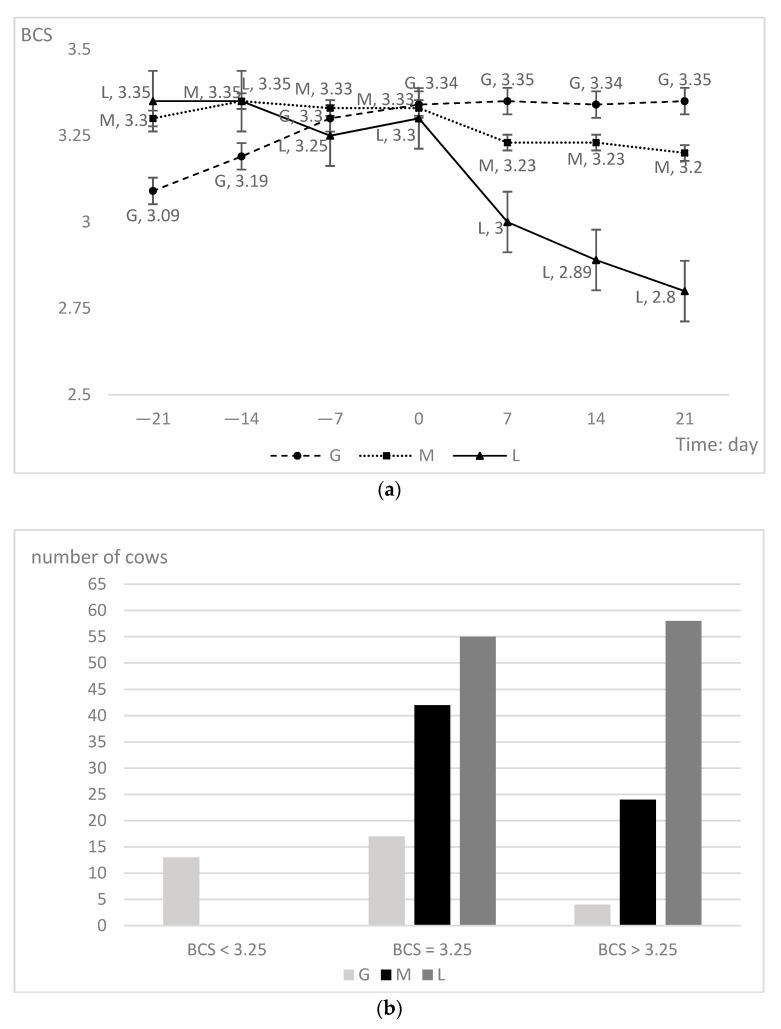
(**a**) Comparison of variation trend of body condition score (BCS) on days 21, 14, 7 before calving, calving day, and 7, 14 and 21 after calving for primiparous cows in different groups. (**b**) Distribution of cows that G (*n* = 34), M (*n* = 66), and L (*n* = 113) groups on −21 days BCS.

The G group experienced a slow rise in the BCS throughout the prepartum period, increasing significantly from −21 days to calving and remaining essentially constant postpartum. Cows that gained BCS had a lower BCS at −21 days. In contrast, the L group had a declining trend over the entire prepartum period: Cows that lost BCS had the highest BCS at the beginning, followed by a slow downward trend and a more dramatic decline after calving. High BCS cows entering the prepartum period had a higher likelihood of losing BCS. This slight decrease was apparent even before calving (Figure 1a). Moreover, when entering the prepartum period, the average BCS of the G group cows was below 3.25. The BCS of the other groups were higher than 3.25. All the BCS were higher than 3.25 at −21 days, except that of the G group. The L group had a higher percentage of cows with BCS greater than or equal to 3.25 (*p* < 0.01; Figure 1b) than the other groups, suggesting that cows with a lower prepartum BCS at −21 days are more likely to have an increased BCS during the prepartum period.

**Figure 2:**
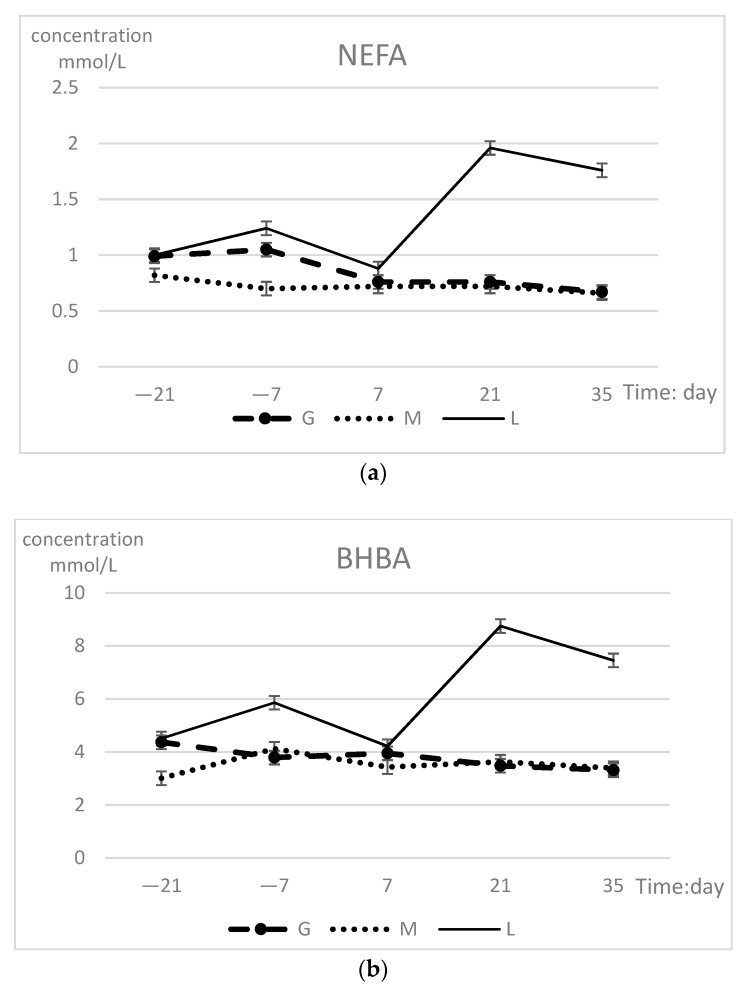
(**a**) Serum NEFA (upper panel) and (**b**) BHBA (lower panel) concentrations (least squares means ± SEM) in different groups during prepartum period.

The NEFA and BHBA concentrations in the three experimental groups are presented in Table 2. The NEFA and BHBA concentrations differed (*p* < 0.01) between groups at postpartum 21 and 35 days. The L group had higher concentrations compared with those in cows that gained or maintained BCS. NEFA and BHBA did not change in a time-dependent manner. There were group-time interaction effects on NEFA (*p* < 0.01) and BHBA (*p* = 0.02). The changes during the prepartum period are shown in Figure 2. The variation trends of the three indicators in the L group had a common feature: The prepartum concentration was slightly higher than that in the other two groups and reached its lowest value on 7 days; then, the concentration sharply increased to significantly higher levels than those of the other two groups, reaching a maximum on 21 days. In the G group, the fluctuations in the prepartum changes were higher than those during postpartum. Cows that maintained BCS showed a smaller change with only slight fluctuations.

**Figure 3:**
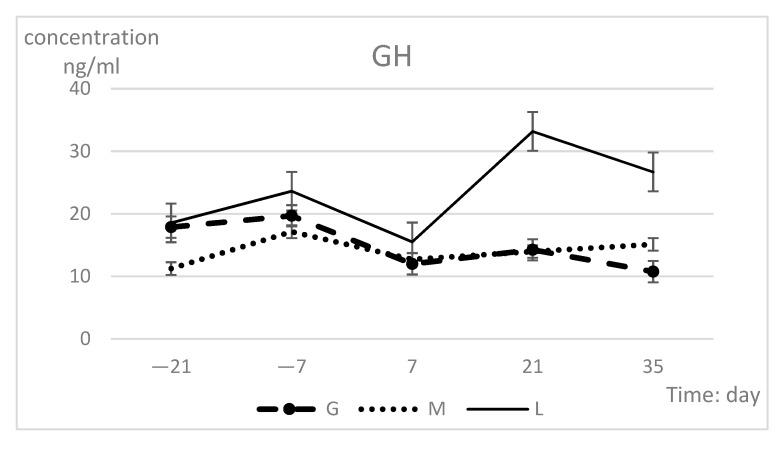

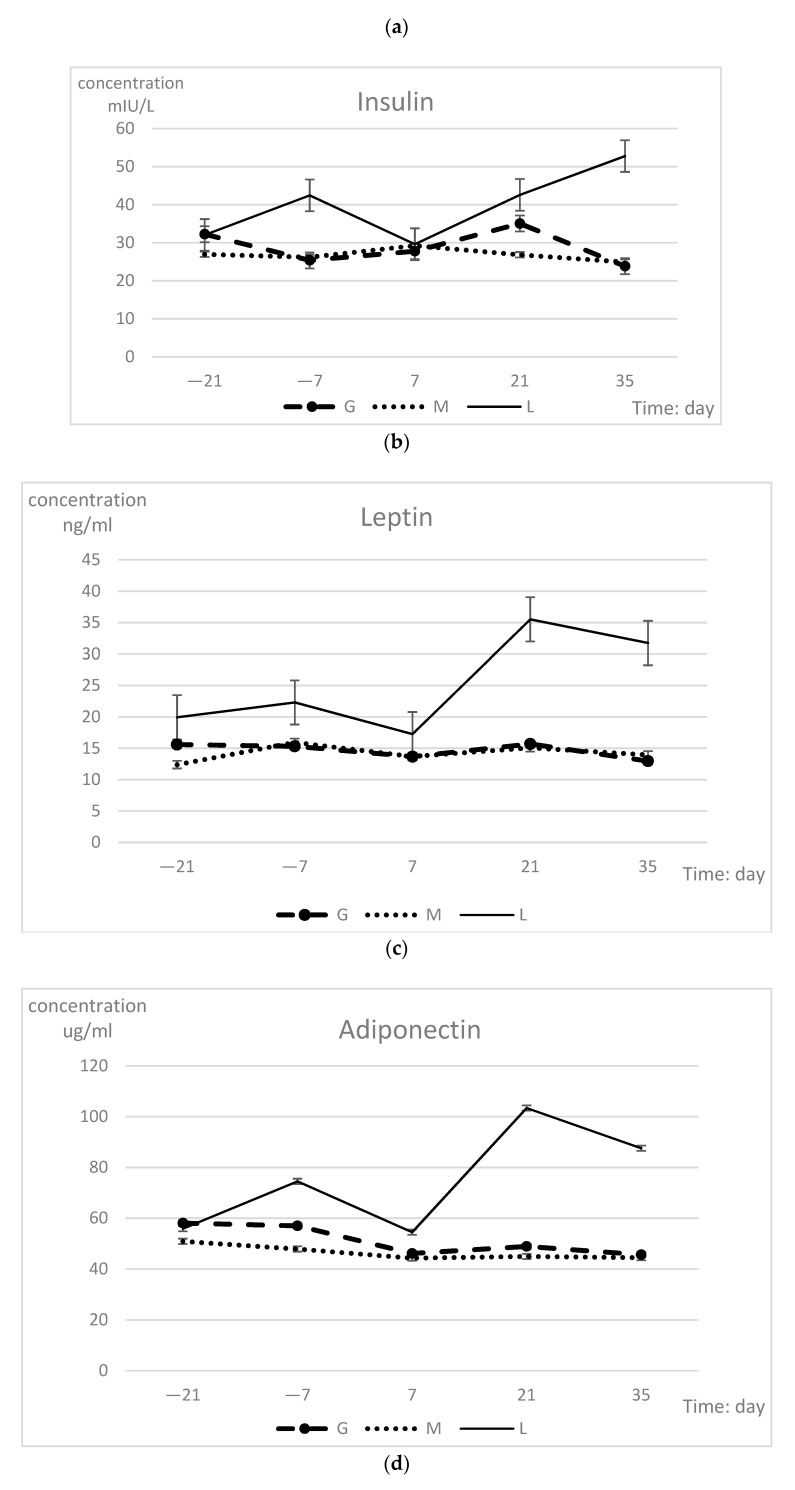
Serum GH (**a**), insulin (**b**), leptin (**c**), and adiponectin (**d**) concentration (least squares means ± SEM) in different groups during prepartum period.

The changes in hormone indexes during peripartum are shown in Table 3. The hormone levels of the groups were highly significantly different (*p* < 0.01), but time had no effect. The GH and adiponectin concentrations exhibited significant difference in group-time interaction (*p* = 0.04). At 21 and 35 days, the L group values were significantly higher than those of the other two groups with regard to GH, leptin, and adiponectin, while the insulin concentrations differed only at 35 days. Each hormone index in the L group showed the most dramatic changes during the prepartum period, with the lowest concentrations observed at 7 days and the highest concentrations at 21 days. The insulin concentrations continued to show an upward trend after 21 days. The GH and insulin indexes of the G group dramatically changed, while the hormone indexes in the M group remained essentially unchanged (Figure 3). The concentrations in all three groups tended to be consistent at 7 days.

The postpartum incidence of disease in cows that gained, maintained, and lost BCS is presented in Table 4. Cows that gained and maintained BCS had fewer health problems than cows that lost BCS. Moreover, when we evaluated cows with one or more health problems, cows that gained and maintained BCS had fewer health events than cows that lost BCS. Milk yield was similar among the experimental groups, and there was no group–time interaction. As reported in Table 5, the cows averaged 24.89 kg/d; however, the time of lactation influenced milk production (*p* < 0.01).

## 4. Discussion

The BCS and its changes are used as indirect indicators to measure fat mobilization and energy balance in individual cows and as a good predictor of the risk of disease. To our knowledge, most studies focus on high-yield multiparous cows, and little is known about the health status, milk yield, hormonal levels, and interrelationships with BCS changes in primiparous cows. A novel contribution of this study is its summary of BCS changes during peripartum in primiparous cows and its emphasis on the effects of varying management of BCS changes on fat mobilization, milk yield, and health between primiparous and multiparous cow. Blood biochemical and hormone indices were analyzed, focusing on primiparous cow management before calving. It is commonly accepted that milk production gradually increases after calving and reaches a peak at approximately 4 weeks postpartum. By contrast, changes in the BCS are inversely related to the lactation curve; however, the use of BCS values as a management tool can be enhanced when the prepartum period is added to the analysis [10,26,27]. Interestingly, it was found that the trend of changes in the prepartum BCS was different from that in the early postpartum lactation period, showing irregular variability. In actual production, it is generally recommended that modern high productivity dairy cows have a moderate BCS (≥3.25 and <3.5) at the beginning of the peripartum period, consequently resulting in lower mobilization of body reserves [28]. Similarly, our results show that primiparous cows with a higher BCS (3.40) at the outset had higher fat mobilization that was specifically reflected in higher NEFA and BHBA concentrations and hormone levels after calving. Surprisingly, the BCS of these cows tended to be consistent at calving, despite the initial variability, implying that the BCS is likely to reach the ideal body condition for calving through prepartum management adjustments, ensuring a suitable BCS prior to calving to minimize fat mobilization.

Ricardo C. et al. (2017) [29] found that BCS changes are strongly related to the BCS at dry off. Dechow et al. (2002) [30] evaluated correlations between the BCS and loss of BCS and found that a higher BCS at calving was phenotypically associated with a higher loss of BCS during early lactation. Our results show that BCS changes during early lactation are largely dependent on the BCS at −21 days, that is, the same as previous finding stated; but have no association to calving BCS because the BCS values at calving were similar between groups. The most important is that the similar BCS for calving comes from changes during prepartum, indicating that change in prepartum BCS plays an important role in the change of early lactation BCS, which determines the change of postpartum. Cows that lost BCS had the highest BCS at −21 days; in contrast, the BCS of cows that gained BCS as lower. This effect is similar to multiparous cows and it is more likely to happen in primiparous cows: Roche et al. (2009) [10] suggested that it is easier for primiparous cows to reach the optimal BCS and have better results in their metabolic and hormonal profiles than multiparous cows. Due to different nutrient partitioning, these cows have a high requirement to maintain good appetite and DMI for continued body growth and development [9,31], leading to a uniform BCS during calving. Moreover, these cows do not go through the drying milk stage and avoid stress caused by changes in feed structure.

Previous studies have confirmed that avoiding BCS loss and maintaining energy balance have positive effects on the fertility, health, and performance of lactating dairy cows. R.V. Barletta reported that a BCS loss was initiated even prior to calving. Our results are clearly consistent with this observation and indicate that BCS changes have an identifiable trend during the prepartum period [32]. The general conclusion is that cows with a relatively consistent BCS exhibit approximately similar changes. The postpartum changes were generally consistent with prepartum changes. The prepartum changes exhibited only a slight change, while the postpartum changes were especially evident in cows that lost BCS. However, there were no significant differences in NEFA, BHBA, or hormone indexes during the prepartum period, indicating that a loss of the prepartum BCS did not involve substantial fat mobilization. Higher circulating concentrations of NEFA, BHBA, and hormones postpartum and a BCS loss happened at the same time, suggesting that primiparous cows are in a parturition stress environment that causes a sharp decrease in DMI, as suggested by Proudfoot et al. (2009) [33]. The majority of studies have reported that DMI is a major driver of variations in the BCS during the early postpartum period. In contrast, insulin and GH are associated with growth [34] and a higher BCS [10], which may explain, in part, the higher concentrations of these metabolites in cows that lost BCS in our study. Additionally, insulin plays a role in energy metabolism, and its concentration is positively correlated with energy intake; however, this relationship has been observed in multiparous cows but not in primiparous cows [8], as confirmed by our experiments.

Usually, circulating NEFA concentrations and DMI have an inverse relationship and are associated with negative effects on health and production. Hayirli et al. (2002) [35] demonstrated that cows classified as obese 21 days before the expected calving date had lower DMI (as a percent of BW, body weight) from 21 days before the expected calving date to calving compared with thinner cows. The reduction in DMI from 21 days before the expected calving date to calving was 40, 29, and 28% for obese, moderate, and thin cows, respectively. In our study, cows that lost BCS had higher levels of NEFA and BHBA than those in the other groups. The effects remained significant after calving, thus confirming that obese cows have low DMI, significant reductions in BCS, and underwent higher fat mobilization after calving. Parity is a well-known pivotal factor for differences in feed intake. Studies have shown that cows with different parity have different characteristics in terms of milk yield, incidence of metabolic disease postpartum, and reproductive performance, with significant differences in feeding habits and behavior during the prepartum period. Primiparous cows have a lower dry matter intake, eat more slowly, are replaced at the feeder more frequently and are typically smaller than multiparous cows. Proudfoot et al. (2009) [33] showed that primiparous cows ate less than multiparous cows during weeks 1–2 after calving. Similarly, H. W. Neave found that even after controlling for BW and milk production, primiparous cows ate less than multiparous cows, with the differences increasing over the postpartum period [36]. Primiparous cows have higher pregnancy rate, lower milk yield, and higher glucose receptor sensitivity than multiparous cows, as well as their physiological functions are vigorous, hormone levels are adequate, there is almost no disorders of glycometabolism and metabolic disease, which is also proven in the study that no ketosis among all the experimental cows. Additionally, cows that lost BCS had higher adiponectin, implying fat mobilization. C. Urh (2018) [37] confirmed the involvement of adiponectin in the regulation of energy partitioning in primiparous cows, with adiponectin concentrations higher than those in multiparous cows. Therefore, nutritional management should be differentiated from multiparous cows that high nutrient and energy concentration to avoid excessive fat in body condition and low DMI postpartum. As for BCS management, it is better to keep a moderate BCS, about 3.25, to enter the peripartum period; higher BCS cows show poor health and performance postpartum, and even a slight drop in the prepartum BCS can be a warning of a postpartum risk of low DIM in primiparous cows. A slight decrease in the prepartum body condition is correlated with a BCS loss after calving and with the magnitude of postpartum NEB.

The increased energy requirements due to lactogenesis and reduced dry matter intake mean that all dairy cows undergo a state of negative energy balance (NEB) from late gestation to early lactation. Additionally, feeding peaks appear after lactation peaks, forcing cows to mobilize their fat reserves or proteins to meet their needs. These problems are increasingly understood to be rooted in DMI 2–3 weeks before calving, arguing for the importance of nutritional management in the prepartum period [38]. Although DMI was not included in this study’s statistics, cows that lost a condition had an increase in leptin, demonstrating that a decrease in DMI leads to lower energy storage levels in the group that lost BCS. At the same time, an increase in the GH concentration meets the higher requirement for body growth and development and fat metabolism. The data indicate that the concentration of insulin increases in cows that lost BCS; however, it is possible that a negative energy balance causes the cows to become insulin resistant, an early warning sign of metabolic disease. One of the purposes of this study was to identify hormone indicators that can efficiently predict changes in the body condition score before calving and can be used as an effective tool for nutritional management; however, these results do not show unified and regular changes in the hormones as a clear means of guiding management.

Our study showed that cows with higher losses of BCS during the peripartum period had higher incidences of abortion, metritis, obstetric canal strain, retention of the fetal membrane, and mastitis during early lactation compared with cows that maintained or gained BCS. Meanwhile, cows that lost BCS had higher risks of lameness and milk fever and were more likely to have more than one health event. In general, cows that maintained BCS had better health than cows that gained or lost BCS. Health status is also associated with elevated concentrations of NEFA and BHBA. An increase in the circulating concentration of NEFA 7 to 10 days prepartum and of BHBA in the postpartum period can indicate metabolic problems, indirectly related to multiple common diseases of energy metabolism [39,40]. Excessive fat mobilization and a long period of NEB can attenuate the function of the immune system [41]. Ospina et al. (2010) [40] demonstrated that an increase in prepartum and postpartum NEFA levels is associated with an increased risk of retained fetal membranes, metritis, clinical ketosis, and displacement of the abomasum. It can be speculated that cows that lost BCS had a negative energy balance and, consequently, an impaired immune response during the periparturient period, possibly predisposing them to metabolic disorders. It should be noted that cows that lost BCS postpartum had higher insulin levels, which is a key adaptive mechanism that raises a concern for the development of insulin resistance and changes in insulin responsiveness [37,42]. Furthermore, primiparous cows experience a suite of stressful events that they have not experienced previously, including regrouping, diet changes, parturition, and the onset of lactation, resulting in reduced food intake and excessive NEB [36].

Intensive genetic selection to increase milk production increases the demands for dietary nutrients and body tissue reserves, resulting in poor health and infertility [43]. The changes in nutrient metabolism required to support lactation in high producing dairy cows are controlled by hormones that coordinate a variety of processes. If the nutritional environment is adequate, cows can meet their energy demands from DMI, and consequently, tissue mobilization will be minimized [10]. Insulin is an especially powerful mediator of a number of various physiological effects, most of which serve to acutely maintain metabolic equilibrium in the face of short-term variations in nutrient supply and demand. Insulin levels were higher after calving in cows that lost BCS: Fewer nutrients are directed to body fat reserves and other non-mammary tissues because of the altered response to insulin, and more nutrients are taken up by the mammary gland consistent with an increase in milk synthesis [44]. However, there were no significant differences in milk production between cows that gained, maintained, or lost BCS. Cows that lost BCS did not have a better production performance.

## 5. Conclusions

In conclusion, it may be easier for primiparous cows to attain an ideal BCS at calving through sufficient prepartum management adjustments, even though a slight drop in the prepartum BCS may constitute a warning of a postpartum risk of large changes in the BCS, greater health problems, and a poor productive performance. Meanwhile, simultaneous increases in hormone levels in cows that lost BCS allows the prevention of excessive lipolysis and metabolic disease, suggesting that their vigorous physiological function and adequate hormone secretion is enough to sustain postpartum demand. In production, it is recommended a moderate BCS of approximately 3.25 for primiparous cows entering peripartum, keeping BCS stable during the prepartum period. Attention should be paid to primiparous cows in the prepartum stage through efficient management, avoiding excessive nutrition, leading to a higher BCS, possibly ensuring higher postpartum DMI and enabling the maximization of potential productivity.

## Figures and Tables

**Table 1:** Comparison of body condition score (BCS; least squares means ± SEM) on days 21, 14, 7 before calving, in relation to calving, and 7, 14, and 21 after calving for primiparous cows in different groups.

Item	Groups			*p*-Value
	G	M	L	
*N*	34	66	113	−
−21 dBCS	3.09 ± 0.06 ^a^	3.39 ± 0.03 ^b^	3.45 ± 0.02 ^b^	<0.01
−14 dBCS	3.25 ± 0.05	3.35 ± 0.03	3.38 ± 0.02	0.19
−7 dBCS	3.30 ± 0.06	3.33 ± 0.04	3.30 ± 0.03	0.33
0 dBCS	3.34 ± 0.07	3.33 ± 0.04	3.28 ± 0.02	0.42
7 dBCS	3.35 ± 0.08 ^a^	3.23 ± 0.05 ^a^	3.00 ± 0.04 ^b^	<0.01
14 dBCS	3.34 ± 0.08 ^a^	3.23 ± 0.05 ^b^	2.89 ± 0.03 ^c^	<0.01
21 dBCS	3.35 ± 0.07 ^a^	3.25 ± 0.05 ^b^	2.8 ± 0.03 ^c^	<0.01

^a–c^ Values within a row with different superscript letters differ at *p* < 0.05. Cows had their BCS evaluated during the transition period (−21 to 21) using a 5-point scale with 0.25 increments. G, gained BCS; M, maintained BSC; L, lost BCS.

**Table 2:** Comparison of NEFA and BHBA contents in different groups of primiparous cattle before and after delivery.

Item	Time	Groups			SEM	*p*-Value		
		G	M	L		Time	Group	G × T
NEFA mmol/L	−21 days	0.99	0.82	1	0.061	0.22	<0.01	<0.01
	−7 days	1.05	0.7	1.24				
	7 days	0.76	0.72	0.88				
	21 days	0.76 ^a^	0.72 ^a^	1.96 ^b^				
	35 days	0.67 ^a^	0.66 ^a^	1.76 ^b^				
BHBA mmol/L	−21 days	4.37	3.01	4.51	0.257	0.21	<0.01	0.02
	−7 days	3.79	4.12	5.86				
	7 days	3.95	3.43	4.22				
	21 days	3.48 ^a^	3.63 ^a^	8.75 ^b^				
	35 days	3.31 ^a^	3.39 ^a^	7.45 ^b^				

^a,b^ Values within a row with different superscript letters differ at *p* < 0.05.

**Table 3:** Comparison of growth hormone (GH), insulin, leptin, and adiponectin contents in different groups of primiparous cattle before and after delivery.

Item	Time	Groups			SEM	*p*-Value		
		G	M	L		Time	Group	G × T
GH ng/mL	−21 days	17.88	11.25	18.55	2.904	0.16	<0.01	0.04
	−7 days	19.69	17.14	23.62				
	7 days	12	12.73	15.51				
	21 days	14.25 ^a^	13.98 ^a^	33.17 ^b^				
	35 days	10.76 ^a^	15.12 ^a^	26.69 ^b^				
Insulin mIU/L	−21 days	32.23	26.95	32.04	1.028	0.09	<0.01	0.07
	−7 days	25.36	26.18	42.45				
	7 days	27.76	29.22	29.61				
	21 days	35.04	26.83	42.58				
	35 days	23.82 ^a^	24.92 ^a^	52.78 ^b^				
Leptin ng/mL	−21 days	15.6	12.39	19.95	1.666	0.77	<0.01	0.32
	−7 days	15.32	15.94	22.31				
	7 days	13.67	13.7	17.26				
	21 days	15.7 ^a^	15.07 ^a^	35.51 ^b^				
	35 days	12.96 ^a^	13.95 ^a^	31.75 ^b^				
Adiponectin ug/mL	−21 days	58.09	50.89	55.9	1.047	0.07	<0.01	0.04
	−7 days	56.99	47.86	74.52				
	7 days	46.15	44.28	54.48				
	21 days	48.91 ^a^	44.97 ^a^	103.41 ^b^				
	35 days	45.65 ^a^	44.45 ^a^	87.59 ^b^				

^a,b^ Values within a row with different superscript letters differ at *p* < 0.05.

**Table 4:** Postpartum incidence (%) of health problems for primiparous cow in different groups.

Item	Groups		
	G	M	L
*N*	34	66	113
Abortion	11.76 (4/34)	4.55 (3/66)	12.39 (14/113)
Metritis		7.58 (5/66)	8.85 (10/113)
Obstetric canal strain			9.73 (11/113)
Retaine fetal membrane	17.65 (6/34)		12.39 (14/113)
Lameness			2.65 (3/113)
Milk fever			11.50 (13/113)
Mastitis		9.09 (6/66)	11.50 (13/113)
Health problems >1			10.62 (12/113)

**Table 5:** Milk yield (least squares means ± SEM) for primiparous cow in different groups.

Item	Groups			*p*-Value		
	G	M	L	Time	Group	G × T
*N*	34	66	113			
Milk yield	24.63 ± 1.65	24.92 ± 1.27	25.12 ± 12.09	<0.01	0.67	0.43
